# Usage, Attitudes, Facilitators, and Barriers Toward Digital Health Technologies in Musculoskeletal Care: Survey Among Primary Care Physiotherapists in Norway

**DOI:** 10.2196/54116

**Published:** 2024-09-16

**Authors:** Lars Martinsen, Nina Østerås, Tuva Moseng, Anne Therese Tveter

**Affiliations:** 1 Center for Treatment of Rheumatic and Musculoskeletal Diseases (REMEDY) Diakonhjemmet Hospital Oslo Norway; 2 Department for Interdisciplinary Health Sciences Faculty of Medicine University of Oslo Oslo Norway; 3 Department of Rehabilitation Science and Health Technology Faculty of Health Sciences Oslo Metropolitan University Oslo Norway

**Keywords:** physiotherapy, physiotherapist, physiotherapists, mHealth, mobile health, app, apps, application, applications, digital health, smartphone, smartphones, ehealth, telemedicine, tele-medicine, family medicine, primary care, primary health care, musculoskeletal, musculoskeletal care, muscle, skeleton, musculoskeletal disorders, MSD, MSDs, internet survey, internet surveys, online survey, online surveys, web-based survey, web-based surveys, survey, surveys, mobile phone

## Abstract

**Background:**

Work burden increases for physiotherapists in the primary health care sector as the prevalence of musculoskeletal disorders (MSDs) increases. Digital health technologies (DHTs) are proposed as a viable solution to secure the sustainability of the health care system and have shown promising results in a range of conditions. However, little is known about use of DHTs among physiotherapists in the primary health care sector in Norway.

**Objective:**

This study aimed to investigate the use of and attitudes toward DHTs among physiotherapists treating patients with MSDs in primary care, and potential facilitators or barriers for adopting DHTs in clinical practice.

**Methods:**

An author-developed web-based questionnaire was distributed to physiotherapists in all Norwegian municipalities in March 2023. The questionnaire included items regarding use of technologies, attitudes, suitability, and factors influencing adoption of DHT. Suitability and agreement on statements were scored on an 11-point numeric rating scale (0=very unsuitable or strongly disagree, 10=very suitable or strongly agree). Differences across employment sites and users versus nonusers of DHT were analyzed using the *χ*^2^ test, Fisher exact test, Student *t* test, and Mann-Whitney *U* test.

**Results:**

Approximately 5000 physiotherapists were invited to participate, of which 6.8% (338) completed the questionnaire. A total of 46.2% (156/338) offered DHTs in their practice, of which 53.2% (83/156) used it on a weekly basis, mostly telephone consultations (105/156, 67.3%). A higher proportion of physiotherapists in private practice offered DHT compared with those employed by municipalities (95/170, 55.9% vs 61/168, 36.3%; *P*<.001). A majority (272/335, 81.2%) were positive about recommending DHTs to their patients. Suitability of DHTs in physiotherapy was rated an average of 6 (SD 2.1). Apps for smartphones or tablets were rated most suitable (mean rating 6.8, SD 2.4). The most frequently reported advantages were flexibility in how physiotherapy is offered (278/338, 82.3%) and reduced travel time for the patient (235/338, 70%). The highest rated disadvantages were limited scope for physical examination (252/338, 74.6%) and difficulty in building rapport with the patient (227/338, 67.2%). The main facilitators and barriers included a functioning (median rating 10, IQR 8-10) or lack of functioning (median rating 9, IQR 8-10) internet connection, respectively. Lack of training in DHTs was prominent regarding evaluation, diagnosing, and treatment (median rating 0, IQR 0-2), with minor, but significant, differences between nonusers and users (median rating 0, IQR 0-1 vs median rating 1, IQR 0-4); *P*<.001).

**Conclusions:**

Physiotherapists in Norwegian primary care treating patients with MSDs are positive about using DHTs, and almost 50% (156/338) have adopted them in clinical practice. Concerns are related to lack of a physical examination and technical aspects. Training in the use of DHTs should be addressed in implementation processes.

## Introduction

The burden of musculoskeletal disorders (MSDs) is high, with an estimated prevalence of 1.7 billion people worldwide [1]. In Norway, 18% of men and 27% of women report chronic MSDs lasting for more than 6 months, and there is an increasing prevalence with age [2]. MSDs account for one-third of all sickness benefits and disability pensions and 9% of all direct health care costs [3]. There is consensus that the majority of these disorders should be treated in primary care [1,4,5]. Among Norwegian patients with MSDs, about 30% have annual contact with primary health care services and 5% to 9% with a physiotherapist [2]. The aging population and expected increase in MSDs threaten the sustainability of the health care system [6,7]. To counteract this unsustainable burden on the health care system, and maintain and improve universal health coverage, increased use of digital health technologies has been suggested as a viable solution [7,8].

Digital health technologies encompass a wide range of different technologies, such as telephone or video consultations, apps, and artificial intelligence [9-11]. Various technologies have already proven to be efficient in the treatment of MSDs [12-15]. However, despite the positive effects, the implementation rate of digital health technologies in physiotherapy practice has been slow [9,16]. Studies during the COVID-19 pandemic in Finland revealed that physiotherapists were largely inexperienced with digital health technologies [17,18]. Several other studies conducted before the COVID-19 pandemic showed similar results; physiotherapists are positive to digital health technologies, but experience barriers to implementing them in clinical practice [19-22].

Despite being one of the most digitalized countries in the Western world [23], little is known about the use of digital health technologies among physiotherapists in primary care in Norway. Given that digital health technologies are highlighted as an important tool in the future of the health care service [7], it is imperative to gain knowledge on physiotherapists’ use of health technologies, their attitudes, and elements relevant for implementation. The overall purpose was to (1) investigate the use of and attitudes toward digital health technologies among physiotherapists treating patients with MSDs in primary care in Norway; (2) explore the suitability, advantages, and disadvantages of digital health technologies in physiotherapy practice; (3) assess potential facilitators and barriers for adopting digital health technologies in clinical practice; and (4) investigate differences in these elements between physiotherapist sector of employment and users versus nonusers of digital health technologies.

## Methods

### Design, Participants, and Recruitment

We used a cross-sectional study design, using an anonymous survey featuring a web-based questionnaire as a method of data collection to answer the research questions. The target population was a convenience sample of physiotherapists actively engaged in the treatment of patients with MSDs and working in primary care in Norway. The study was conducted in accordance with the STROBE (Strengthening the Reporting of Observational Studies in Epidemiology) statement [24] and CHERRIES (Checklist for Reporting Results of Internet E-Surveys) [25].

The survey was limited to physiotherapists treating adults with MSDs, defined as conditions sorted under chapter L in the Norwegian version of the *International Classification of Primary Care, 2nd edition* (*ICPC-2*) [26]. Participants were informed regarding the scope of the survey and limitations in participation through the information letter following the link to the survey. No stratification on specialty or rostering at the independent employment site was induced. As no distinct definition of digital health technologies exists, based on previous descriptions we defined digital health technologies as digital methods or tools used in the evaluation, diagnosis, and treatment of patients with MSDs, in in-patients, out-patients, and remote settings [8,16]. We excluded the use of electronic health records and digital communication between health care personnel from our definition, as this is ubiquitous in the Norwegian health care service.

The physiotherapy service in primary care in Norway is organized as a combination of municipality-employed physiotherapists on a fixed salary and physiotherapists in private practice. Municipality-employed physiotherapists typically work in an in-patient or home-based setting, either alone or as a part of a multidisciplinary team. Physiotherapists in private practice usually work in an out-patient physiotherapy clinic, either with an operating grant to practice within the municipality, or solely on the cost of the patient. There are no clear guidelines determining which patients should receive physiotherapy from a municipality-employed physiotherapist or a physiotherapist in private practice. However, differences in the characteristics of the patients may occur, and we therefore analyzed these sectors separately in our study.

The survey was distributed as an open survey collecting anonymous data. An email with an invitation to participate was sent on March 20, 2023, to either the head of the physiotherapy service or a shared email address for official correspondence in all the 356 Norwegian municipalities. The email contained a link to the survey through a digital solution. The email was also sent directly to physiotherapists whose email addresses were available on the municipalities’ websites. This included physiotherapists working in private practice. In addition, the link to the questionnaire was advertised on social media (Facebook [Meta] and Twitter [rebranded as X]) by the authors and in their networks. The questionnaire was open for response for 2 weeks and closed on April 3, 2023. No incentive to participate was granted to the responders.

### The Questionnaire

The questionnaire was developed based on previous questionnaires covering similar topics [18,27-30], and discussion within the members of the research group. The research group included 4 physiotherapists with extensive experience with digital health technologies in musculoskeletal physiotherapy and clinical experience as physiotherapists in primary care, and 2 patient research partners.

Nettskjema, a web-based survey tool by the University of Oslo, was used to construct the questionnaire [31]. Access to the questionnaire was only possible through a unique link, leading directly to the survey. The questionnaire included 49 items, divided into 12 questions, 37 statements, and additional free-text fields (not analyzed in this study). The selection of items was guided by the Unified Theory of Acceptance and Use of Technology (UTAUT), which posits that an individual’s intention to use an information system can be explained by 4 key constructs, that are performance expectancy, effort expectancy, social influence, and facilitating conditions [32]. A mandatory employment question determined access to the rest of the questionnaire, allowing only physiotherapists reporting to work in primary care to proceed. Further questions covered the demographic characteristics of the participants (sex, age, and work experience), and the use, recommendation, and suitability of digital health technologies. The type of digital health technologies and frequency of use were conditionally displayed only to those stating to be offering digital health technologies. Statements covered attitudes toward using digital health technologies, and facilitators and barriers toward adopting such technologies. Suitability and agreement on statements were scored on an 11-point numeric rating scale (0=very unsuitable or strongly disagree and 10=very suitable or strongly agree). A score of ≥7 on attitudes was considered a positive attitude [33]. Items were distributed on 8 electronic pages, with 1 to 12 items per page. Except for the question on employment in primary care, no questions were mandatory. No completeness check was therefore provided; however, the responders had the opportunity to review and check their answers before submitting the questionnaire. Nettskjema does not provide information on the number of views or the participation rate. As no cutoff for minimum completion of the questionnaire was applied in this study, the completion rate is similar to users who agreed to participate.

The questionnaire was pretested in a sample of 3 independent physiotherapists with experience in primary care and treatment of MSDs, and the members of the research group, including the patient research partners. Improvement of accuracy and clarity, including adding questions and statements regarding reimbursement and data security, was subsequently implemented in the questionnaire.

### Statistical Method

Stata version 17 (StataCorp) was used for all analyses and the significance level was set at *P*<.05. Noncontinuous variables are presented as frequency counts and percentages, whereas continuous variables are presented as mean values. Differences between employment sites and users or nonusers of digital health technology in usage, recommendation, advantages, and disadvantages were analyzed using the chi-square test. Where expected values were <5, the Fisher exact test was used. Similarly, to assess differences in suitability, facilitators, barriers, and attitudes, Student *t* test was used. Data were visually inspected for normality by assessing histograms and quantile-quantile plots. If normality was not met in analyses of facilitators, barriers, and attitudes, the Mann-Whitney *U* test was conducted. Wilcoxon signed-rank test was conducted for assessing differences in attitudes regarding the evaluation, diagnosing, and treatment of acute and chronic conditions. Questionnaires with more than 50% missing items were removed. No imputation was performed for missing values. No cut-off point for atypical timestamps was induced, and neither were any corrections to adjust for nonrepresentative samples. Of the physiotherapists in private practice, 16 worked without operating grants. These 16 did not differ from the physiotherapists with operating grants on any aspects in this survey, and the 2 categories were merged to 1.

### Ethical Considerations

The study was conducted in line with The Declaration of Helsinki [34]. As no health information and only anonymous data were processed in this survey, ethical approval from the National Research Ethics Committees was not required. This is in accordance with the Norwegian Health Research Act and The Personal Data Act including the General Data Protection Regulation (GDPR) [35]. However, an assessment of the privacy of the questionnaire was undertaken by the Institutional Data Protection Officer and an Institutional Board at Diakonhjemmet Hospital before the data collection. Information regarding the purpose of the study, privacy, institutional affiliation, principal investigator, and consent to publication was included in the web questionnaire and provided to all participants before answering.

## Results

### Demographics

An estimated 5000 physiotherapists were invited to participate in the survey, of which 338 (6.8%) completed the questionnaire. The majority were female (226/338, 66.9%), and the mean age was 43.4 (SD 11.1) years (Table 1). The responders were equally divided between municipality-employed physiotherapists (168/338, 49.7%) and physiotherapists in private practice (170/338, 50.3%). A large majority had more than 10 years of work experience. Only 1 questionnaire was removed due to more than 50% missing values.

**Table 1:** Characteristics of responders.

		Total group (N=338)	Employed by municipalities (n=168)	Private practice (n=170)
Age (years), mean (SD)		43.4 (11.1)	40 (10.5)	46.7 (10.7)
Sex (female), n (%)		226 (66.9)	138 (82.1)	88 (51.8)
**Work experience, n (%)^a^**				
	Less than 1 year	9 (2.7)	9 (5.4)	0 (0)
	1-5 years	38 (11.3)	28 (16.9)	10 (5.9)
	6-10 years	42 (12.5)	33 (19.9)	9 (5.3)
	More than 10 years	246 (73.5)	96 (57.8)	150 (88.8)

^a^Total group (n=335), employed by municipalities (n=166), private practice (n=169).

### Use of Digital Health Technologies

Digital health technologies were offered by 46.2% (156/338) of the physiotherapists. A significantly higher proportion of physiotherapists in private practice (95/170, 55.9%) offered digital health technology compared with those employed by municipalities (61/168, 36.3%; χ^2^_1_=13, *P*<.001). More than half of those who offered digital health technologies used it on a weekly basis (Table 2), with a significantly higher frequency of use observed among physiotherapists in private practice compared with those employed by municipalities . Only 10.2% (16/156) used digital health technologies daily. No differences in the use of the various technologies were found, except from telephone and video consultations, which were significantly more frequently used among physiotherapists in private practice compared with municipality-employed physiotherapists.

A large majority of the physiotherapists (272/335, 81.2%) were positive to recommending the use of digital health technologies to patients with MSDs. Significantly higher proportions of municipality-employed physiotherapists were positive compared with physiotherapists in private practice (144/166, 86.8% vs 128/169, 75.7%; χ^2^_1_=6.6*, P*=.01), as well as physiotherapists offering digital health technologies compared with physiotherapists not offering (143/155, 92.3% vs 129/180, 71.7%, χ^2^_1_=23.1, *P*<.001).

**Table 2:** Use of digital health technologies.

		Total group (n=156), n (%)	Employed by municipalities (n=61), n (%)	Private practice (n=95), n (%)	Chi-square (*df*)		*P* value	
**Frequency of use**						14.2 (4)		.005^a^
	Never	5 (3.2)	3 (5)	2 (2)				
	1-2 times a month	68 (43.6)	36 (59)	32 (34)				
	Once a week	28 (18)	10 (16)	18 (19)				
	3-5 times a week	39 (25)	10 (16)	29 (30)				
	Every day	16 (10.2)	2 (4)	14 (15)				
**Digital health technologies offered**								
	Telephone consultations	105 (67.3)	35 (57)	70 (74)	4.5 (1)		.03	
	Apps for smartphones or tablets	103 (66)	46 (75)	57 (60)	3.9 (1)		.047	
	Video consultations	63 (40.4)	17 (28)	46 (48)	6.5 (1)		.01	
	Activity trackers	15 (9.6)	5 (8)	10 (11)	0.2 (1)		.63	
	Gaming	4 (2.6)	2 (3)	2 (2)	0.2 (1)		.64^a^	
	Virtual reality	2 (1.3)	0 (0)	2 (2)	1.3 (1)		.52^a^	
	Augmented reality	1 (0.6)	0 (0)	1 (1)	0.6 (1)		.99^a^	
	Artificial intelligence	1 (0.6)	1 (2)	0 (0)	1.6 (1)		.39^a^	
	Robotics	1 (0.6)	0 (0)	1 (1)	0.6 (1)		.99^a^	

^a^Fisher exact test.

### Suitability of Digital Health Technologies

A mean score of 6 (SD 2.1) was reported on the overall suitability of digital health technologies in physiotherapy practice with a significant, but small difference, between municipality-employed physiotherapists and physiotherapists in private practice. Similarly, significant but small differences were found regarding the suitability of apps for smartphones or tablets, activity trackers, games, and artificial intelligence, with municipality-employed physiotherapists more positive about the suitability of all technologies, except video consultations (Table 3). Overall, the therapists already offering digital solutions rated suitability significantly higher on all solutions compared with those not offering digital solutions (results not shown).

**Table 3:** Suitability of digital health technologies.

Digital health technology			Suitability^a^										
			Total group, mean (SD)		Employed by municipalities, mean (SD)		Private practice, mean (SD)		Mean difference (95% CI)		*t* test (*df*)		*P* value
Overall			6 (2.1)		6.3 (1.8)		5.7 (2.3)		0.6 (0.1 to 1.0)		2.6 (331)		.01
**Specific**													
	Apps for smartphones or tablets	6.8 (2.4)		7.2 (2.0)		6.5 (2.6)		0.7 (0.2 to 1.2)		2.8 (332)		.006	
	Video consultations	6.1 (2.4)		6.2 (2.2)		6.1 (2.6)		0.1 (–0.4 to 0.6)		0.3 (331)		.74	
	Activity trackers	5.8 (2.5)		6.2 (2.4)		5.5 (2.5)		0.7 (0.01 to 1.2)		2.3 (327)		.02	
	Telephone consultations	5.4 (2.6)		5.2 (2.5)		5.6 (2.7)		–0.4 (–0.1 to 0.1)		–1.5 (332)		.13	
	Robotics	4.9 (2.8)		5.1 (2.8)		4.6 (2.8)		0.5 (–0.1 to 1.1)		1.6 (316)		.11	
	Virtual reality	4.8 (2.6)		5.1 (2.5)		4.6 (2.7)		0.5 (–0.1 to 1.1)		1.7 (319)		.09	
	Augmented reality	4.7 (2.7)		4.9 (2.6)		4.4 (2.7)		0.5 (–0.1 to 1.1)		1.6 (316)		.10	
	Gaming	4.7 (2.7)		5.2 (2.7)		4.2 (2.6)		1.0 (0.4 to 1.6)		3.3 (317)		.001	
	Artificial intelligence	4.2 (2.7)		4.5 (2.6)		3.8 (2.8)		0.7 (0.1 to 1.2)		2.1 (310)		.04	

^a^0=very unsuitable, 10=very suitable.

### Advantages and Disadvantages of Digital Health Technologies

Digital health technologies’ contribution to flexibility in how physiotherapy is offered was agreed upon by 82.3% (278/228) of the responders. As well, reduction in travel for patients (235/338, 69.5%) and improved access (207/338, 61.2%) were highlighted as advantages. Where significant differences were found, the municipality-employed physiotherapists consistently responded more positively to the statements compared with the physiotherapists in private practice (Table 4).

Regarding disadvantages with digital health technologies, the limited scope for physical examination (252/338, 74.6%) and difficulty in building a rapport with the patient (227/338, 67.2%) were the two most frequently reported. A significant difference was found regarding low digital competence of the patients, with a greater proportion of municipality-employed physiotherapists reporting this as a disadvantage as compared with physiotherapists in private practice (Table 5).

**Table 4:** Advantages of digital health technology.

Advantage	Total group, n (%)	Employed by municipalities, n (%)	Private practice, n (%)	Chi-square (*df*)	*P* value
Offers flexibility in how physiotherapy is delivered	278 (82.3)	148 (88.1)	130 (76.5)	7.8 (1)	.005
Reduction in travel for the service user	235 (69.5)	134 (79.8)	101 (59.4)	16.5 (1)	<.001
Improved access to physiotherapy	207 (61.2)	114 (67.9)	93 (54.7)	6.2 (1)	.01
Modernizes our approach to communication	179 (53)	85 (50.6)	94 (55.3)	0.7 (1)	.39
More efficient for conducting and attending meetings	169 (50)	100 (59.5)	69 (40.6)	12.1 (1)	<.001
Less time consuming than conventional interventions	153 (45.3)	100 (59.5)	53 (31.2)	27.4 (1)	<.001
Good service user’s satisfaction	123 (36.4)	55 (32.7)	68 (40)	1.9 (1)	.17
Useful for continuing your professional development	80 (24)	39 (23)	41 (24)	0.04 (1)	.85
Reduces “did not attend” rate	71 (21)	38 (23)	33 (20)	0.5 (1)	.47
Good job satisfaction for the physiotherapist	54 (16)	22 (13)	32 (19)	2.1 (1)	.15
Adequate to outrule serious pathologies	18 (5)	7 (4)	11 (7)	0.9 (1)	.35
An adequate subjective and objective examination can be completed	15 (4)	7 (4)	8 (5)	0.06 (1)	.81
No advantages	13 (4)	3 (2)	10 (6)	3.8 (1)	.049

**Table 5:** Disadvantages of digital health technology.

Disadvantage	Total group, n (%)	Employed by municipalities, n (%)	Private practice, n (%)	Chi-square (*df*)	*P* value
Limited scope for the physical examination	252 (74.6)	130 (77.4)	122 (71.8)	1.4 (1)	.24
Difficult to build a rapport with the service user	227 (67.2)	116 (69.1)	111 (65.3)	0.5 (1)	.46
Computer literacy of the service user is poor	211 (62.4)	133 (79.2)	78 (45.9)	39.9 (1)	<.001
Inadequate ability to rule out serious pathologies	201 (59.5)	106 (63.1)	95 (55.9)	1.8 (1)	.18
Difficult to alleviate service user’s concerns regarding their health	159 (47)	83 (49.4)	76 (44.7)	0.7 (1)	.39
Difficult to communicate “bad news” to the service users	126 (37.3)	59 (35.1)	67 (39.4)	0.7 (1)	.41
Reduces service user satisfaction	89 (26)	43 (26)	46 (27)	0.1 (1)	.76
The technology will fail regularly	73 (22)	45 (27)	28 (17)	5.3 (1)	.02
Difficult to prescribe a specialized treatment plan	58 (17)	27 (16)	31 (18)	0.3 (1)	.60
Difficult to ensure privacy and confidentiality	53 (16)	31 (19)	22 (13)	1.9 (1)	.16
Reduces job satisfaction for the physiotherapist	38 (11)	14 (8)	24 (14)	2.8 (1)	.09
Difficult to obtain consent	31 (9)	23 (14)	8 (5)	8.2 (1)	.004
More time consuming that conventional interventions	20 (6)	7 (4)	13 (8)	1.8 (1)	.18
Increases “did not attend” rate	16 (5)	7 (4)	9 (5)	0.2 (1)	.63
No disadvantages	7 (2)	1 (1)	6 (4)	3.6 (1)	.06^a^

^a^Fisher exact test.

### Facilitators and Barriers

**Figure 1:**
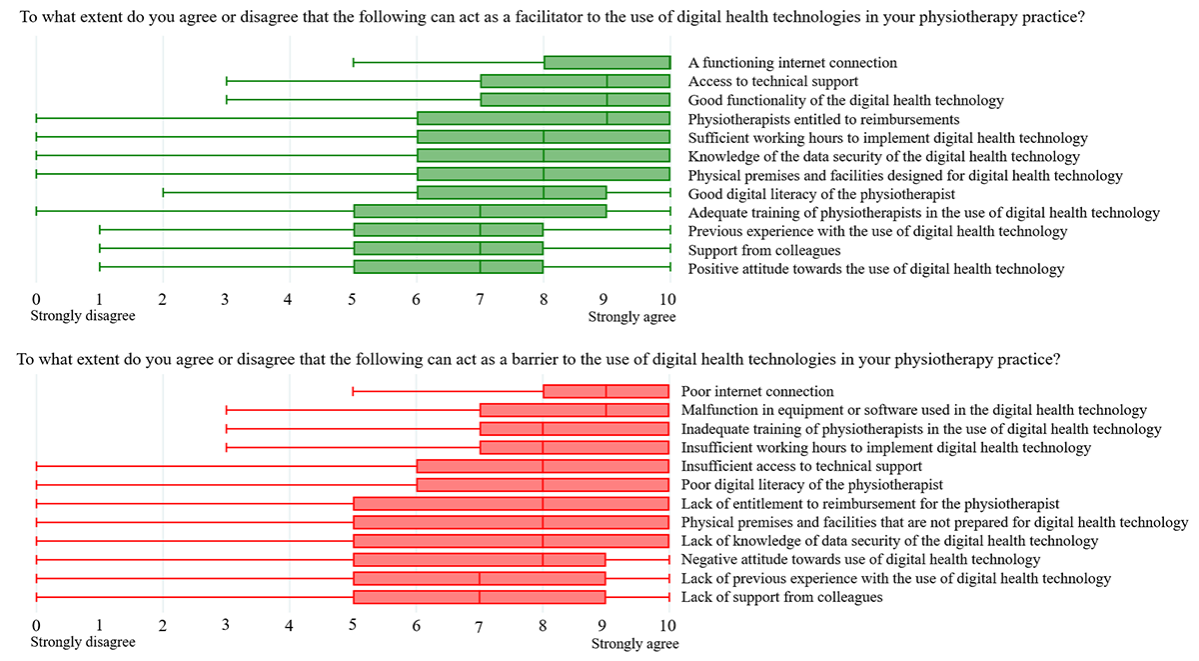
Facilitators and barriers to adopting digital health technologies.

Among facilitating factors, technical aspects of using digital health technologies showed high median scores, especially a functioning internet connection (median 10, IQR 8-10) and access to technical support (median 9, IQR 7-10). Similarly, lack of technical infrastructure showed high median scores as barriers to adopting digital health technologies, with a poor internet connection (median 9, IQR 8-10) and malfunction in equipment or software used in the digital solution (median 7, IQR 7-10) ranking as primary barriers (Figure 1). Minimal differences were found between municipality-employed physiotherapists and physiotherapists in private practice in their responses to factors acting as facilitators or barriers. The physiotherapists in private practice reported entitlement to reimbursement as significantly more important both as a facilitator (median 10, IQR 7.5-10 vs median 8, IQR 5-10; *z*=–4.3; *P*<.001) and a barrier (median 9, IQR 6-10 vs median 8, IQR 5-10; *z*=–3.2; *P*=.002) as compared with the municipality-employed physiotherapists. However, municipality-employed physiotherapists rated the importance of all other statements regarding facilitators and barriers higher than physiotherapists in private practice.

### Attitudes Toward Digital Health Technologies

The physiotherapists expressed a significantly higher confidence in treating patients using digital health technologies compared with evaluating and diagnosing, both in acute (median 4, IQR 2-6 vs median 3, IQR 2-5; *z*=–5.3; *P*<.001) and chronic conditions (median 5, IQR 3-7 vs median 4, IQR 2-6; *z*=–7.2; *P*<.001). In addition, they reported a significantly higher confidence in evaluating and diagnosing patients with chronic conditions compared with acute conditions (median 4, IQR 2-6 vs median 3, IQR 2-5; *z*=–2.6; *P*=.01), and similar for treatment (median 5, IQR 3-7 vs median 4, IQR 2-6; *z*=–3.9; *P*<.001). Physiotherapists offering digital health technologies were significantly more confident in using technologies in evaluating, diagnosing, and treating patients both regarding acute and chronic conditions compared with those not offering them. A large majority of the responders disagreed that they had received training in using digital health technologies. Although both groups disagreed with the statement, a significant difference was observed between those offering digital health technologies and those who did not; with those not offering such technologies scoring significantly lower than those who offered the technologies (Multimedia Appendix 1).

## Discussion

### Principal Findings

In this study, we found that the use of digital health technologies was approximately 50% among the responders, with a higher frequency of use among physiotherapists in private practice. The suitability of digital health technologies was rated as high, with the municipality-employed physiotherapists scoring suitability more positively, together with those already using digital health technologies. The advantages of digital health technologies reflected benefits for both patients and the physiotherapists, however, the lack of physical examination was a prominent disadvantage. The municipality-employed physiotherapists appeared as more positive toward the advantages of digital health technologies, and as more confident regarding its use. Technical aspects could serve as both facilitators and barriers to adopting the technology. Our study is believed to be the first study quantifying the extent of use of digital health technologies among this group of physiotherapists, and one of the first assessing facilitators and barriers in a postpandemic setting.

As waiting lists and the prevalence of MSDs increase, the health care sector will be dependent on adopting digital health technologies in the future [36]. Limitations in access to trained health care personnel, supplied by an expectation of high-quality care from the patients, will entail a shift in the provision of care in a direction of using more digital health technologies [7]. Our data demonstrate that approximately half of the physiotherapists in our study already used digital health technologies on a weekly basis. A rapid increase of technology has previously been observed during the COVID-19 pandemic [33,37,38], and it appears that the use of technology has been sustained for some of the physiotherapists. Prominent in our data was the use of telephone and video in consultations as well as the use of apps for smartphones and tablets. Our results are in line with the results in the Norwegian national eHealth survey, stating that the preferred digital contact with patients among health care personnel is telephone consultations, followed by written digital contact and video consultations [39]. Notable in our study is the very low use of other technologies—technologies that we could expect to be relevant in the treatment of patients with MSDs. Gaming and virtual reality have shown beneficial effects in other patient populations, including increasing physical performance, that potentially could have been used in musculoskeletal physiotherapy [40-42].

The large majority of physiotherapists were positive toward recommending digital health technologies to patients with MSDs. Together, with the positive view of the suitability of digital health technologies in physiotherapy, this indicates that there is a potential for increased use of such technologies within primary care physiotherapy for patients with MSDs. The most frequently reported advantages of digital health technologies contained benefits both for patients and the physiotherapists, reflecting its potential in increasing accessibility and reducing barriers to treatment for the patients. Interestingly, nearly half of the respondents (153/338, 45.3%) stated that an advantage of digital health technology is its reduced time consumption compared with conventional interventions. On the other hand, the limited scope for physical examination (252/338, 74.6%) and difficulty in building a rapport with the patient (227/338, 67.2%) were noted as disadvantages of the technology. It is assumed that this reflects the nature of physiotherapy, a profession that traditionally has relied on a hands-on approach [43]. The latter is most likely also reflected in the difference found between using digital health technology in evaluating and diagnosing, compared with treatment. The increased confidence in using technology in treatment likely reflects that the physiotherapists feel dependent on a physical meeting to perform an adequate clinical evaluation, a concern reported by physiotherapists in previous literature [30,44,45]. However, studies have found high levels of agreement between face-to-face and telehealth evaluations, somewhat in contradiction to the expressed disadvantage in our study [46,47]. Resistance toward changing practice has been found to be a barrier for implementing digital health technologies among health care professionals in previous studies, but whether this expressed dependence on a physical examination reflects such a resistance is unclear [48].

An interesting finding in our data is that the physiotherapists using digital health technologies were more positive toward its suitability. Furthermore, they reported a higher confidence in using the technology in physiotherapy practice. These findings are in line with evidence provided in other research works [21,49]. Given the notable result in our data regarding the lack of training in the use of digital health technologies, it may not come as a surprise that the physiotherapists more experienced in using the technology had a more positive attitude. Hellstén et al [50] suggest that this indicates a “learning by doing” approach by the physiotherapists and that the experience of the physiotherapist may affect their use. However, as both the users and nonusers expressed a lack of training, the latter should be an aspect of concern if increased use of digital health technology is warranted. Providing proper training is essential to overcome a range of previously expressed barriers, such as lack of digital literacy among the health care personnel, concerns regarding diminished patient safety, and issues with securing privacy and confidentiality of health information [51-53].

A magnitude of factors that could act as facilitators and barriers for implementing digital health technologies in the health care sector has been found in previous studies. From an organizational perspective, the cost of implementing digital solutions is often cited as a barrier, especially highlighting the lack of systems regarding reimbursement for digitally provided care [37,51]. This was also found in our study. The physiotherapists in private practice, who receive their payment as a combination of reimbursement and deductible from the patients, emphasized entitlement to reimbursement as a significantly more important facilitator and barrier than the municipality-employed physiotherapists. In a health care system like the Norwegian, which to a certain degree relies on a combination of health care personnel on a fixed salary and on reimbursement, this is an important aspect. It is unlikely that a further change in clinical care toward adopting digital health technologies will continue only based on the goodwill of the physiotherapists and without certain financial incentives.

The main facilitators and barriers found in our study were related to technology. Similar results have been found in other studies, with technological aspects serving as both a facilitator [50,54] and a barrier [49,55]. The degradation of physiotherapist-related aspects, and the emphasis on aspects regarding technology and infrastructure, might indicate that a further adoption of digital health technologies is affected by implemented measures on a system level. However, there is a lack of research regarding the optimal integration of technology into the health care sector on a system level [56]. To reach the full potential of digital health technologies, further research on the implementation and integration of technology in the digital ecosystem should be prioritized.

We also noted some differences between the municipality-employed physiotherapists and physiotherapists in private practice in our results. An overall finding is that the municipality-employed physiotherapists, though having less frequent use of the technology, appeared more positive about its use and suitability. In addition, the municipality-employed physiotherapists generally scored both advantages and disadvantages, and the importance of the facilitators and barriers, higher than the physiotherapists in private practice. The frequency of use of digital health technologies was higher among physiotherapists in private practice, including significantly more frequent use of telephone and video consultations. This likely reflects a difference in clinical practice as physiotherapists in private practice predominantly practice in out-patient clinics, whereas municipality-employed physiotherapists tend to conduct home visits and provide services in nursing homes and rehabilitation facilities more frequently. Also, patient characteristics could differ as municipality-employed physiotherapists predominantly treat an older patient population, characterized by a higher proportion of diagnoses related to geriatrics, functional deterioration, and fall, rather than MSDs [57]. Previous studies have shown that age and general digital skills are closely linked [58]. In our study, the municipality-employed physiotherapists reported low digital competence of the patients as a disadvantage in relation to digital health technology to a significantly higher degree compared with the physiotherapists in private practice. This could possibly indicate that there is an age difference in the patient population between the practices.

### Strengths and Limitations

There are some limitations to our study. Despite distributing the survey to all Norwegian municipalities, we only had a response rate of approximately 7%. However, the response rate is comparable with other countries [18], and our study includes more than twice as many responding physiotherapists as in the Norwegian national eHealth survey [39]. Distributing the survey to physiotherapists in all municipalities in Norway has most likely given us a nationwide representativity, covering both rural and urban areas. An almost 50/50 distribution between municipality-employed physiotherapists and physiotherapists in private practice increases the generalizability of the study. Caution should be exercised in generalizing the study findings beyond a Norwegian primary care setting and physiotherapists treating patients with MSDs. The limitation to primary care was chosen as this will be an important setting for treating patients with MSDs in the future and the limitation to MSDs was made to improve interpretability of the results. These limitations might influence the response rate and our results, as we are not certain whether we have captured all aspects of the use of digital health technologies among Norwegian physiotherapists. A volunteer bias may exist in our study, as the therapists with less positive experience and impression of digital health technologies may have refused to respond. The questionnaire was based on consensus in the research group, drawing on previous literature and questionnaires and pretesting; however, the lack of a standardized questionnaire could be a limitation. While it is recommended to include a mix of positively and negatively worded questions to avoid response bias [59], our questionnaire included only positively worded questions as we were cautious about altering the existing questionnaires. Web-based data collection secured a widespread distribution of the questionnaire. Due to the anonymous nature of the questionnaire and no option for cookies or IP checks in the Nettskjema tool, multiple entries may have been possible, although we believe that this is unlikely.

### Conclusion

Almost 50% of physiotherapists in Norwegian primary care treating patients with MSDs have adapted the use of digital health technologies, particularly those in private practice. The physiotherapists expressed positive attitudes to the use of digital health technologies, and more so if they already offered it. However, challenges in adapting technologies included the need for a physical examination to exclude severe pathology and in-person meetings to establish a relationship, which appear as the greatest disadvantages. Technical aspects and an appropriate scheme for reimbursement served as both facilitators and barriers. Notably, lack of training in the use of digital health technologies was prominent and appeared as a barrier and should likely be addressed in future research and implementation.

## Appendix

Multimedia Appendix 1 Attitudes toward digital health technologies.

## Abbreviations

CHERRIES: Checklist for Reporting Results of Internet E-Surveys
GDPR: General Data Protection Regulation
ICPC-2: International Classification of Primary Care, 2nd edition
MSD: musculoskeletal disorder
STROBE: Strengthening the Reporting of Observational Studies in Epidemiology
UTAUT: Unified Theory of Acceptance and Use of Technology
