# Supplementary material for: Usage, Attitudes, Facilitators, and Barriers Toward Digital Health Technologies in Musculoskeletal Care: Survey Among Primary Care Physiotherapists in Norway
Source: JMIR Rehabil Assist Technol. 2024 Sep 16;11:e54116. doi: 10.2196/54116 (PMC11443180; doi:10.2196/54116)
Supplement: Multimedia Appendix 1 [file rehab_v11i1e54116_app1.pdf]

Multimedia Appendix 1: Attitudes towards digital health technologies

This is a Multimedia Appendix to a full manuscript published in the JMIR Rehabilitation and Assistive Technologies. For full copyright and citation information see http://dx.doi.org/10.2196/54116.

	Total group, mean (SD)	Not offering technology, median (IQR)	Offering technology, median (IQR)	*z, P*-value[a]
I am confident I can use digital health technologies to evaluate and diagnose acute MSDs	3 (2-5)	3 (1-5)	5 (3-6)	-5.465, *P*<.001
I am confident I can use digital health technologies to treat acute MSDs	4 (2-6)	3 (2-5)	5 (4-7)	-5.372, *P*<.001
I am confident I can use digital health technologies to evaluate and diagnose chronic MSDs	4 (2-6)	3 (2-5)	5 (3-6)	-4.160, *P*<.001
I am confident I can use digital health technologies to treat chronic MSDs	5 (3-7)	5 (2-6)	5 (4-7)	-3.416, *P*<.001
I have all the required resources to use digital health technologies to manage patients with MSDs	2 (0-4)	1 (0-3)	3 (1-6)	-5.244, *P*<.001
Using digital health technologies to manage patients with MSDs is part of my professional role	3 (2-6)	2 (0-5)	5 (3-7)	-7.262, *P*<.001
I know how to effectively diagnose and treat effectively via digital health technologies	3 (1-5)	1 (0-3)	4 (2-6)	-7.958, *P*<.001
Using digital health technologies to manage MSD patients is as effective as face to face care	2 (0-4)	1.5 (0-3)	3 (1-5)	-4.978, *P*<.001

Patients value treatment with digital health technologies to the same extent as face to face care	2 (1-5)	2 (0-3)	3 (2-5)	-6.191, *P*<.001
I have been trained in the use of digital health technologies for the assessment and diagnosis of MSDs	0 (0-2)	0 (0-1)	1 (0-3)	-5.156, *P*<.001
I have been trained in the use of digital solutions for the treatment of MSDs	0 (0-2)	0 (0-1)	1 (0-4)	-5.826, *P*<.001

0=strongly disagree, 10=strongly agree. [a]= Mann-Whitney U test
